# Relationship between social support, functional outcomes and health-related quality of life in working-aged adults at three months after ischemic stroke: results from the FRAILTY study

**DOI:** 10.1186/s12955-025-02337-3

**Published:** 2025-01-27

**Authors:** Elena Gurková, Daniela Bartoníčková, Daniel Šaňák, Šárka Šaňáková, Jana Zapletalová, Lenka Štureková

**Affiliations:** 1 Department of Nursing, Faculty of Health Sciences, Palacký University, Olomouc, Czech Republic; 2 Comprehensive Stroke Center, Department of Neurology, Palacký University Medical School and Hospital, Hněvotínska 3, Olomouc, 775 15 Czech Republic; 3 Department Medical Biophysics, Faculty of Medicine and Dentistry, Palacký University, Olomouc, Czech Republic

**Keywords:** Ischemic stroke, Health-related quality of life, Social support, Functional outcomes, Working age

## Abstract

**Background:**

The relationship between social support and functional outcomes and health-related quality of life (HRQoL) after ischemic stroke (IS) remains unclear, especially in working-aged patients.

**Aim:**

To assess the relationship between perceived social support, functional outcomes, post-stroke psychosocial symptoms, and HRQoL in working-aged adults three months after IS.

**Methods:**

A prospective and correlational design was used. Patients of working age (18–65 years) admitted for first-ever IS were enrolled in the prospective FRAILTY (Factors Affecting the Quality of Life After Ischemic Stroke in Young Adults) study (NCT04839887). HRQoL (using the Stroke Impact Scale, Quality of Life in Neurological Disorders), social support (using the Multidimensional Scale of Perceived Social Support), functional outcomes (using a modified Rankin Scale—mRS), and post-stroke psychosocial symptoms were assessed three months after IS. Descriptive statistics, Wilcoxon signed-rank test, Spearman’s correlations and multiple linear regression were used for analysis.

**Results:**

A total of 121 (54.5% males, mean age 51.7 ± 8.4 years) IS patients were analyzed. Of those, 87.7% had excellent clinical outcomes (mRS 0–1) after three months. Patients reported significant improvement in all domains of self-reported HRQoL except memory and communication after three months. The overall perceived social support was not associated with HRQoL domains. Post-stroke depression was negatively associated with all domains of HRQoL. Living arrangements (living alone) and post-stroke depression were negatively associated with perceived social support after IS.

**Conclusions:**

More insight into the relationship between variables of HRQoL in working-aged adults might increase their social participation, strengthen supportive relationships, and promote their recovery and rehabilitation process. Focusing on the management of emotional problems and supporting functional outcomes may be modifiable factors that may represent targets for strategies to improve the HRQoL. Further research is needed to clarify the relationship between pre-stroke perceived social support and its types and post-stroke psychosocial symptoms in the long term.

## Background

Currently, there is an emerging trend of increasing ischemic stroke (IS) in younger adults, as recently noted by epidemiologists [1]. A broad spectrum of sociodemographic, clinically related (stroke severity, functional outcomes, fatigue, aphasia; [2–6], and psychosocial variables (post-stroke depression and anxiety, cognitive dysfunction, fear from stroke recurrence, social support and social network, and return to work; [3, 6–11] have been examined as factors contributing to health-related quality of life (HRQoL) in working-aged adults after IS [12]. Social support and social networks have been recognized as independent predictors of HRQoL after IS, as they can help reduce the physical and psychosocial sequelae of IS [13]. Several theoretical models have been developed to explain the effect of social support, such as the ‘main effect model’ and the ‘stress-buffering model’ [13].

Patients after IS are at risk of loneliness and losing contact with friends and social activities [14, 15], and significant associations between social support and HRQoL have been found previously [2, 16–18]. Social support is crucial for working-aged IS patients with regard to their specific needs (rehabilitation goals, early return to work) and key familial and social roles [19]. However, healthcare professionals usually do not consider or acknowledge these needs sufficiently [19, 20].

Despite extensive evidence about the role of social support during the first months after a first-ever stroke [2, 21–25], only few studies have included young stroke patients under 65 years of age [7, 18]. Most previous studies have focused on the social consequences of stroke, and examined return to work as a proxy for stroke recovery or stroke rehabilitation outcomes. The social consequences of IS in younger patients also include the impact on spousal and family relationships (disruption of family relations), parenting [26], individual/family finances, or social participation (reduced involvement in leisure and other social activities; [23, 27].

Previous qualitative studies have highlighted spousal and parental tasks [12, 28, 29], feelings of stigma and shame [30], or perception of invisible and not well accepted impairments in social, community and work contexts [31]. Quantitative studies [6, 13, 23] have reported inconsistent evidence on the impact of social support on HRQoL after IS, and only one cross-sectional study has examined this relationship in young adults [6].

The present study aimed to assess the possible relationship between social support, functional outcomes, post-stroke psychosocial symptoms, and HRQoL in working-aged adults three months after IS. The findings of our study may help explore a possible impact of social support on HRQoL, post-stroke psychosocial symptoms (depression and anxiety, cognitive function), and recovery after IS in the specific stroke population of working-aged patients.

## Methods

### Study sample

Consecutive IS patients enrolled in the longitudinal observational cohort study FRAILTY (Factors Affecting the Quality of Life After Ischemic Stroke in Young Adults; ClinicalTrials.gov identifier NCT04839887, registered on April 9, 2021) between April 2022 and April 2024 were analyzed.

The FRAILTY study was approved by the Ethics Committee of the University Hospital Olomouc and the Faculty of Medicine and Dentistry, Palacký University Olomouc (June 2021, no. NU22-09–00021). All enrolled patients gave signed informed consent.

The inclusion criteria were as follows: age 18–65 years; hospital admission for first-ever IS confirmed by computed tomography or magnetic resonance imaging of the brain; ability to understand the content of the questionnaires and to communicate and sign informed consent. The exclusion criteria were as follows: transient ischemic attack without progression to IS; cerebral infarction caused by trauma; hemorrhagic stroke; history of cognitive impairment or communication disorder resulting in impaired ability to understand the questionnaires; concurrent severe systemic illness that could affect HRQoL.

The sample size was calculated in G*Power 3.1 program a priori for multiple linear regression analysis: fixed model, R^2^ increase (F-test). Assuming a medium effect size of 0.15, a significance level of 0.05, a minimum accepted power of 0.80, and 11 predictors for multiple regression, the minimum required sample size was 123 respondents [32].

#### Data collection

The following groups of variables based on Wilson and Cleary’s conceptual model of HRQoL adapted for stroke survivors [6] were assessed before discharge from a stroke unit and three months after discharge: (1) individual variables (age, sex, education, marital status, number of children, household/living arrangements, use of health services, return to work); (2) stroke-related physical and functional variables (residual neurological deficit, post-stroke pain, functional status or disability); (3) cognitive function; (4) psychosocial factors (post-stroke depression, post-stroke anxiety); and (5) domains of HRQoL.

Neurological deficit at admission and after three months was scored using the National Institute of Health Stroke Scale (NIHSS), and functional outcomes after three months using the modified Rankin Scale (mRS) and Barthel index (BI). Excellent functional outcome was defined as mRS 0–1 and a BI of 100 points. The Montreal Cognitive Assessment (MoCA; [33]) was used as a standard screening tool for global cognition comprising several domains (e.g., visuospatial abilities, memory, attention, orientation) with a recommended cut-off of 26 for cognitive impairment.

### Patient-reported outcomes at baseline and after a three-month follow-up: psychosocial sequelae and HRQoL

#### Post-stroke pain

The Czech version of the Brief Pain Inventory (BPI; [34]), which assesses clinical pain, was used to rate the severity and degree to which pain interferes with typical dimensions of feeling and function. The BPI includes items on the site of pain, the severity of pain (pain at its worst in the past 24 h, pain at its least in the past 24 h, pain on average, and pain right now), and how pain interferes with the patient’s mood, walking ability, normal work, relations with other people, sleep, and enjoyment of life. These items are rated on a scale of 0 to 10: a higher score means more significant pain or more interference with the above activities.

### Psychological factors – post-stroke depression and anxiety

To assess anxiety and depression after IS, a self-report screening scale, the Czech version Hospital Anxiety and Depression Scale (HADS; [35]), was used. The HADS contains 14 items measuring depression (HADS-D, seven questions) and anxiety (HADS-A, seven questions). Each item has four response options, with a possible score of 0–3. The total score for each scale is 0–21 points. A cut-off score of 8 for mild depression/anxiety and a score of 11 for definite depression/anxiety (11 to 14, moderate; 15 to 21, high) is considered the cut-off point.

### Psychosocial sequelae of IS and HRQoL

Specific HRQoL after IS was assessed with the Czech version of the Stroke Impact Scale (SIS), version 3.0 [36]. The scale, designed to assess multidimensional stroke outcomes, includes the following eight dimensions: strength (4 items), hand function (5 items), activities of daily living / instrumental activities of daily living (ADL/IADL, 10 items), mobility (9 items), communication (7 items), emotion (9 items), memory and thinking (7 items), and participation/role function (8 items). Scores are calculated by generating a summative transformed score for each domain (ranging from 0 to 100), with higher scores indicating a lower impact of stroke on HRQoL. In addition, an extra single-item domain includes a visual analog scale from 0 to 100 and focuses on stroke recovery assessment. The instrument is recommended for longitudinal monitoring of changes after stroke in research or clinical practice.

To measure psychosocial sequelae of IS and HRQoL specific for adults with neurological disorders, the Czech version of the Quality of Life in Neurological Disorders (Neuro-QoL; [37, 38]) was used. The Neuro-QoL is a set of 13 brief self-report measures. The following Czech versions of Neuro-QoL measures were used in the study: v2.0 – ability to participate in social roles and activities; v1.0 – anxiety; v1.0 – depression; v1.0 emotional and behavioral dyscontrol; v1.0 – fatigue; v1.0 – lower extremity function – mobility; v1.0 – sleep disturbance; v1.0 – upper extremity function – fine motor; v1.0 – satisfaction with social roles and activities; v1.0 – cognitive function; v1.0—stigma; and v1.0 – positive affect and well-being. According to the scoring manuals, the total scores of the neuro-QoL patient-reported outcomes are calculated and converted to the corresponding T-scores. Each scale ranges from 0 to 100, where 50 represents the mean of the reference population. One standard deviation is 10 points, and a change of 0.5 SD is considered meaningful.

### Social support

The Czech version of the Multidimensional Scale of Perceived Social Support (MSPSS; [39]) was used to assess perceived social support after IS. The MSPSS is the most widely used one to measure social perception provided by an individual's perception of social support from three sources: social support from family, friends, and significant others. It includes 12 items divided into three subscales addressing a different source of support (social support from family, friends, and significant others). The MSPSS has been validated as a three-factor model (social support from family, friends, and significant others) using different target groups, most frequently chronic patients. Higher scores indicate higher levels of perceived social support. Each subscale is summed and divided by four, and all the items are summed and divided by 12 to provide the total score.

### Use of health services

The use of health care services during a three-month follow-up period (FUP) after discharge was studied by items developed by authors. The health or community-based services involved nursing home care agencies, physiotherapists, speech therapists, occupational therapists, psychological care, psychiatric care, social workers, and nutrition therapy. The type and frequency of each service used during the FUP and its availability were recorded. Two 5-point Likert-type scales were assigned to each item. The first scale evaluated the frequency of use, and the second one the availability of individual health services used. Higher scores indicated greater frequency and availability. Patients stated for each item how often they use each type of health service and at the same time evaluated how this type of health service is accessible for them.

### Return to work

Return to work (RTW) in the FUP was assessed in a semi-structured interview with open-ended questions during an outpatient clinical examination after three months. The interview guide was discussed and revised in cooperation with patients after IS. The interview guide consists of open-ended questions focusing on current occupation, occupation/workplace/working hours before and after stroke, experiences of RTW after stroke (circumstances affecting return process, functioning in work nowadays, changes in occupation/workplace/working hours before and after stroke etc.). The responses were categorised into 3 themes: RTW without changing occupational status, RTW with changing occupational status, not RTW.

### Data analysis

The study data were analyzed using the IBM SPSS Statistics, Version 23.0 (Armonk, NY: IBM Corp.), with a statistical significance level set at 0.05. The Shapiro–Wilk test assessed the normality of data distribution. The data were analysed using descriptive and inferential statistics. The Wilcoxon signed-rank tests were used to examine changes in perceived social support, stroke-related clinical variables (stroke severity, functional outcomes, cognitive functions), psychosocial sequelae of stroke and HRQoL (domains of the SIS 3.0) in three months of FUP.

Spearman’s correlations and multiple linear regression analyses were used to explore the relationships between independent and dependent variables. A multiple linear regression analysis was conducted to identify predictors of stroke impact in young stroke patients and to investigate the relationship between HRQoL (operationalized SIS 3.0 domains) three months FUP (dependent variables) and the following independent variables: demographic factors factors (age, gender, living arrangements, marital status, total number of children); stroke-related factors (severity of stroke operationalized by the NIHSS total score before the discharge); functional status or disability (measured by the mRS before the discharge); baseline MoCA; MSPSS Total score three months FUP; post-stroke depression three months FUP (operationalized by the HADS-depression total score) and anxiety three months FUP (the HADS-anxiety total score). Only variables correlated with domain scores were entered into individual models for predicting SIS domain scores. The number of variables entering the multiply linear regression models is closely related to the sample size requirements. In regression based on the Enter method, there should be at least 20 cases for each variable [40].

To identify significant predictors of the MSPSS Total score after three months, a multiple linear regression analysis was performed with independent variables: HADS anxiety and HADS depression after three months of follow-up, age, and living arrangements. These were the ones that correlated in the first stage of the correlation analysis.

Preliminary tests of the regression model matrix included the assessment of normality, linearity, multicollinearity, homoscedasticity, independence of residuals, and outliers. The multicollinearity of variables was measured by correlation coefficients, variance inflation factor (VIF), and the tolerance index before the regression analysis. If the tolerance index is 0.2 or less, or VIF is greater or equal to 5, multicollinearity exists in the data. The relationships between variables showed homoskedasticity, i.e., homogeneity of variance. Durbin–Watson statistic was used to detect the presence of autocorrelation in the residuals (prediction errors) from regression analyses. The Durbin-Watson statistic lies in the range 0–4. A value of 2 or nearly 2 indicates no first-order autocorrelation. An acceptable range is 1.50—2.50. Where successive error differences are small, Durbin-Watson is low (less than 1.50), indicating positive autocorrelation. The Durbin-Watson statistic was low (1.148) only in regression analysis of social support predictors, suggesting positive autocorrelations. Selected demographic (living arrangements – living alone / living with someone; sex – male / female; age – ≤ 50 years / > 50 years.; total number of children—without categorization) and clinical variables (NIHSS, mRS, MoCA score) were dichotomized for purposes of regression analysis.

## Results

**Fig. 1:**
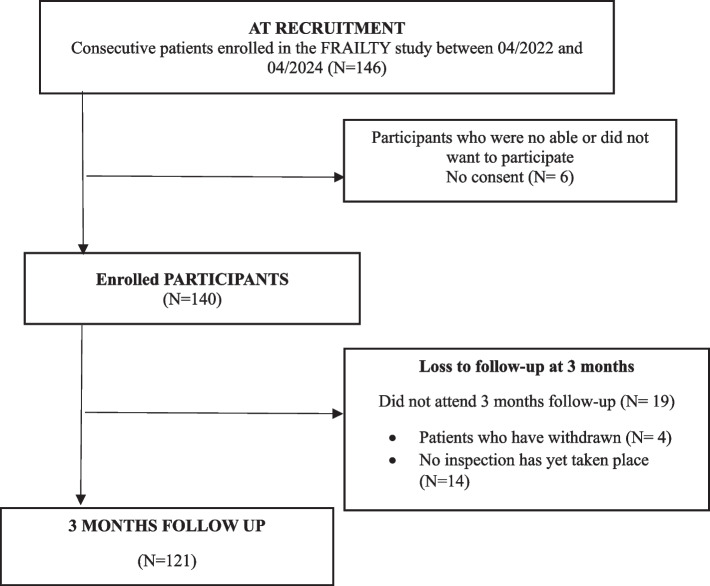
Study flowchart of patients' enrolment for the study analysis

The demographics, baseline, and clinical characteristics of participants are shown in Table 1. In a 2-year time frame, 146 patients met the inclusion criteria, and 140 (95.8%) consented to participate. At 3-month follow-up, 121 (85.2%) completed the measures (Fig. 1). After three months, one hundred and five (87.6%) patients had excellent clinical outcomes (mRS 0–1). There were 54.5% male respondents. Up to 50 years were included 41.3% of the respondents. Most of the respondents had Apprenticeship training (44.6%), followed by secondary education (33.1%). Most of the respondents lived with spouse/partner (72.7%) and were married (55.4%). Before stroke, 89.3% had a full-time job. Return to work without changing occupational status was reported by 56.2% of participants, and 15.7% reported RTW with changing occupational status. The use of health care services during a three-month follow-up period was diverse. However, only a minority reported using physiotherapy services after discharge from hospital (17.6%).

**Table 1:** Selected demographic, baseline and clinical characteristics of all enrolled patients

Patient characteristics	Value
Age—mean (± SD, years)	51.7 (± 8.4)
IS in anterior circulation – n (%)	98 (80.3)
IV thrombolysis—n (%)	62 (50.8)
Mechanical thrombectomy – n (%)	40 (32.9)
Arterial hypertension—n (%)	86 (70.5)
Ischemic heart disease—n (%)	9 (7.3)
Diabetes mellitus—n (%)	12 (9.8)
Atrial fibrillation—n (%)	14 (11.5)
Hyperlipidemia—n (%)	90 (73.8)
Smoking—n (%)	52 (42.7)
NIHSS and mRS score 0 at 90 days after IS—n (%)	94 (77.7)
NIHSS 1 at 90 days after IS- n (%)	9 (6.0)
mRS 1 at 90 days after IS- n (%)	12 (9.9)
NIHSS 2 at 90 days after IS- n (%)	8 (6.6)
mRS 2 at 90 days after IS- n (%)	10 (8.3)
NIHSS > 2 at 90 days after IS- n (%)	9 (6.0)
mRS > 2 at 90 days after IS- n (%)	5 (4.1)
BI score at 90 days after IS—median (range)	100 (70–100)
MoCA score at 90 days after IS – median (range)	26 (13–30)

*BI* Barthel index, *IS* ischemic stroke, *IV* intravenous, *mRS* modified Rankin Scale, *NIHSS* National Institutes of Health Stroke Scale, *SD* standard deviation

### Changes in perceived social support, stroke-related clinical variables, and psychosocial sequelae of stroke in three months follow up

After three months (Table 2), a significant improvement in MoCA score and HRQoL (except Memory and Communication dimensions) was observed. There was a significant decrease in HADS depression and anxiety (Table 3). However, no changes in the MSPSS scale (social support evaluation) were observed during the FUP (Table 3).

**Table 2:** Stroke severity, functional outcomes, and cognitive function at baseline and after the three-month FUP

**Variable**	**Mean**	**SD**	**Median**	**Minimum**	**Maximum**	p-value
NIHSS, baseline	1.8	2.5	1.0	0	14	** < 0.0001**
NIHSS, 90 days after IS	0.5	1.2	0.0	0	6	
NIHSS, difference	−1.0	2.0	−1.0	−10	6	
mRS, baseline	1.1	1.3	1.0	0	5	** < 0.0001**
mRS, 90 days after IS	0.4	0.8	0.0	0	4	
mRS, difference	−1.0	1.0	0.0	−4	2	
MoCa, baseline	25.1	4.9	26.0	13	30	** < 0.0001**
MoCa, 90 days after IS	27.2	2.9	28.0	13	30	
MoCa, difference	2.0	4.0	2.0	−6	26	
**SIS 3.0 dimension**						
Strength, baseline	69.6	27.1	75.0	0	100	** < 0.0001**
Strength, 90 days after IS	81.7	19.8	87.5	25	100	
Strength, difference	13.6	27.5	6.3	−44	100	
Memory, baseline	86.2	17.5	92.9	11	100	0.304
Memory, 90 days after IS	88.8	15.2	92.9	14	100	
Memory, difference	3.9	21.8	0.0	−46	100	
Emotion, baseline	69.7	16.9	72.5	25	100	**0.018**
Emotion, 90 days after IS	73.9	16.3	77.5	25	100	
Emotion, difference	4.1	17.8	2.5	−40	63	
Communication, baseline	88.6	19.8	100.0	11	100	0.074
Communication, 90 days after IS	93.1	10.6	100.0	46	100	
Communication difference	5.6	21.1	0.0	−29	100	
ADL/IADL, baseline	85.2	25.0	97.5	0	100	**0.0001**
ADL/IADL, 90 days after IS	93.5	12.4	100.0	33	100	
ADL/IADL, difference	9.3	22.0	0.0	−35	95	
Mobility, baseline	83.6	26.4	95.8	0	100	** < 0.0001**
Mobility, 90 days after IS	92.8	12.3	100.0	44	100	
Mobility, difference	10.4	24.3	0.0	−50	94	
Hand function, baseline	79.3	31.0	100.0	0	100	** < 0.0001**
Hand function, 90 days after IS	88.6	22.1	100.0	0	100	
Hand function, difference	10.3	24.4	0.0	−40	100	
Social participation, baseline	72.3	29.3	81.3	0	100	**0.021**
Social participation, 90 days after IS	77.7	23.8	81.3	3	100	
Social participation, difference	5.6	27.3	3.1	−84	91

**Table 3:** Social support, post-stroke depression and anxiety at baseline and after the three-month FUP

**Variable**	**Mean**	**SD**	**Median**	**Minimum**	**Maximum**	p-value
MSPSS total, baseline	6.21	1.27	6.58	1	11	0.217
MSPSS total, 90 days after IS	6.18	0.98	6.42	2	7	
MSPSS total, difference	−0.063	1.215	0.000	−5.17	5.17	
MSPSS significant others, baseline	6.35	1.24	7.00	1	7	0.484
MSPSS significant others, 90 days after IS	6.40	0.91	6.75	2	7	
MSPSS significant others, difference	0.022	1.160	0.000	−4.00	5.75	
MSPSS family, baseline	6.21	1.29	6.75	1	7	0.899
MSPSS family, 90 days after IS	6.27	1.19	6.75	2	7	
MSPSS family, difference	0.031	1.210	0.000	−4.83	5.50	
MSPSS friends, baseline	6.06	1.83	6.25	1	21	0.762
MSPSS friends, 90 days after IS	6.07	1.15	6.25	2	7	
MSPSS friends, difference	−0.010	1.956	0.000	−15.00	5.75	
HADS anxiety, baseline	1.30	0.60	1.0	1	3	** < 0.0001**
HADS anxiety, 90 days after IS	0.40	0.70	0.0	0	2	
HADS anxiety, difference	−0.90	0.80	−1.00	−3	1	
HADS depression, baseline	1.30	0.50	1.0	1	3	** < 0.0001**
HADS depression, 90 days after IS	0.40	0.70	0.0	0	2	
HADS depression, difference	−0.90	0.70	−1.00	−3	1	

Patients statistical significantly increased the scores of the following SIS 3.0 domains: Strength, Emotion, ADL, Mobility, Hand function, and Social participation (Table 2). Clinically meaningful changes were found only in domains of Social Participation, Strength, and Emotions. In the present study, we considered it to be a positive clinically meaningful change in scores when the difference in domain score was + 15 points or more; no change when the difference was between −14 and + 14; and a negative clinically meaningful change when the difference in score was −15 or lower [35].

### Correlation and regression analyses between perceived social support, stroke-related clinical variables, HRQoL and psychosocial sequelae of IS

Social support from family, friends, and other familiars was significantly correlated with emotional aspects of HRQoL (SIS 3.0 and NeuroQoL), stigma (Neuro-QoL Stigma), cognitive functions (Neuro-QoL Cognitive functions) and social participation (SIS 3.0) and following domains of NeuroQoL: 'Satisfaction with Social Roles and Activities', 'Ability to Participate in Social Roles and Activities' (Table 4). However, these correlations ranged from weak to moderate. This study also aimed to determine the most important predicting variables that explain the largest proportion of variance of HRQoL. The detailed results are shown in the Table 5. The overall MSPSS score did not predict any HRQoL domain (Table 5). These results suggest that HRQoL is not strongly associated with perceived social support in working-age adults after stroke.

**Table 4:** Spearman correlation coefficients (r) between MSPSS and HRQoL (SIS 3.0; Neuro-QoL), HADS, BPI

	**MPSPSS Total scale**	**MSPSS –** **Significant others**	**MSPSS – Family**	**MSPSS – Friends**
**SIS 3.0**				
SIS—Strength	0.151	0.147	0.103	0.126
SIS—Memory	0.280 **	0.278 **	0.285 **	0.250 **
SIS – Emotion	0.441 ***	0.471 ***	0.399 ***	0.393 ***
SIS – Communication	0.246 **	0.262 **	0.246 **	0.245 **
SIS – ADL	0.231 *	0.105	0.161	0.281 **
SIS – Mobility	0.240 **	0.152	0.228 **	0.255 **
SIS – Hand function	0.126	0.117	0.098	0.179
SIS – Social participation	0.240 **	0.128	0.245 **	0.245 **
Neuro-QoL Anxiety T score	−0.308 ***	−0.313 ***	−0.264 **	−0.282 **
Neuro-QoL Depression T Score	−0.389 ***	−0.370 ***	−0.367***	−0.362 ***
Neuro-QoL Fatigue T Score	−0.276 **	−0.212 *	−0.246 **	−0.239 **
Neuro-QoL Stigma T Score	−0.358 ***	−0.314 ***	−0.355 ***	−0.380 ***
Neuro-QoL Sleep Disturbance T Score	−0.153	−0.131	−0.163	−0.174
Neuro-QoL Satisfaction with Social Roles and Activities T Score	0.250 **	0.184 *	0.217 *	0.256 **
Neuro-QoL Ability to Participate in Social Roles and Activities T Score	0.389***	0.316 ***	0.340 ***	0.408 ***
Neuro-QoL Lower Extremity Function – Mobility T Score	0.267 **	0.259 **	0.234 **	0.297 ***
Neuro-QoL Positive Affect and Well-Being T Score	0.461***	0.431 ***	0.409 ***	0.451 ***
Neuro-QoL Upper Extremity Function – Fine Motor T Score	0.249 **	0.169	0.159	0.312 ***
Neuro-QoL Cognitive functions T Score	0.341 ***	0.345***	0.343***	0.334***
Neuro-QoL Emotional and Behavioral Dyscontrol T Score	−0.291***	−0.369***	−0.260	−0.248
**HADS**				
HADS -Anxiety	−0.321***	−0,258**	−0.270**	−0.281**
HADS—Depression	−0.406 ***	−0,409 ***	−0.405 ***	−0.381 ***
**BPI**				
BPI Severity	−0.058	−0.141	−0.049	0.002
BPI Interfeence	−0.131	−0.190	−0.139	−0.092

^*^p< 0.05

**p< 0.01

***p< 0.001

**Table 5:** Predictors of HRQoL (operationalised SIS 3.0 domains) after three months. Results of multivariate linear regression analysis (model Enter)

**Dependent variable: Overall recovery** (ANOVA p < 0.0001); Adjusted R^2^: 0.478						
	**Coefficient B**	**SE**	**Standardized coefficients β**	**95%CI for B**	**t-value**	**p-value**
Constant	86.8	2.96		80.93–92.68	29.30	< 0.0001
HADS-anxiety 90 days after IS	0.876	0.439	1.193	0.005–1.748	1.99	0.049
HADS-depression 90 days after IS	−2.523	0.435	−0.564	−3.385 to −1.166	−5.80	< 0.0001
NIHSS above 8, baseline	−13.03	8.149	−0.125	−29.19 to 3.133	−1.599	0.113
mRS 3–5, baseline	−14.37	4.366	−0.267	−23.03 to −5.712	−3.29	0.001
MoCA above 26, baseline	9.734	2.915	0.249	3.953–15.52	3.34	0.001
**Dependent variable: Strenght** (ANOVA p < 0.0001); Adjusted R^2^: 0.413						
	**Coefficient B**	**SE**	**Standardized coefficients β**	**95%CI for B**	**t-value**	**p-value**
Constant	88.30	3.09		82.18–94.42	28.6	< 0.0001
HADS-anxiety 90 days after IS	0.576	0.466	−0.127	−1.500 to 0.348	−1.236	0.219
HADS-depression 90 days after IS	−1.301	0.462	−0.290	−2.218 to −0.384	−2.81	0.006
NIHSS above 8, baseline	−12.40	8.668	−0.118	−29.59 to 4.788	−1.431	0.156
mRS 3–5, baseline	−12.68	4.635	−0.234	- 21.87 to −3.493	−2.74	0.007
MoCA above 26, baseline	11.07	3.074	0.282	4.969–17.163	3.60	0.0005
**Dependent variable: Memory** (ANOVA p < 0.0001); Adjusted R^2^: 0.346						
	**Coefficient B**	**SE**	**Standardized coefficients β**	**95%CI for B**	**t-value**	**p-value**
Constant	99.20	1.71		95.81–102.6	58.20	< 0.0001
HADS-anxiety 90 days after IS	−0.209	0.336	−0.066	−0.876 to 0.458	−0.622	0.535
HADS-depression 90 days after IS	−1.570	0.331	−0.499	−2.226 to 0.915	−4.75	< 0.0001
Living arrangement (alone)	−9.352	2.966	−0.244	−15.23 to −3.473	−3.15	0.002
**Dependent variable: Emotions** (ANOVA p < 0.0001); Adjusted R^2^: 0.475						
	**Coefficient B**	**SE**	**Standardized coefficients β**	**95%CI for B**	**t-value**	**p-value**
Constant	88.6	I.78		85.11–92.17	49.8	< 0.0001
HADS-depression 90 days after IS	−1.509	0.345	−0.412	−2.192 to −0.825	−4.374	< 0.0001
HADS-anxiety 90 days after IS	−1.215	0.351	−0.327	−1.911 to −0.519	−3.463	0.001
Living arrangement (alone)	−6.459	3.095	−0.145	−12.59 to −0.324	−2.087	0.039
**Dependent variable: Communication domain** (ANOVA p < 0.0001); Adjusted R^2^: 0.231						
	**Coefficient B**	**SE**	**Standardized coefficients β**	**95%CI for B**	**t-value**	**p-value**
Constant	84.8	6.27		72.36–97.24	13.5	< 0.0001
HADS-anxiety 90 days after IS	−0.040	0.279	−0.017	−0.592 to 0.513	−0.142	0.887
HADS-depression 90 days after IS	−0.789	0.293	−0.331	−1.370 to −0.209	−2.69	0.008
MPSS Total	1.722	0.928	0.167	−0.117 to 3.561	1.856	0.066
MoCA above 26, baseline	4.15	1.752	0.200	0.672–7.618	2.37	0.020
**Dependent variable: ADL domain** (ANOVA p < 0.0001); Adjusted R^2^: 0.502						
	**Coefficient B**	**SE**	**Standardized coefficients β**	**95%CI for B**	**t-value**	**p-value**
Constant	99.4	1.853		95.68–103.03	53.6	< 0.0001
HADS-anxiety 90 days after IS	0.227	0.280	0.077	−0.328 to 0.782	0.812	0.419
HADS-depression 90 days after IS	−1.238	0.278	−0.423	−1.789 to −0.687	−4.459	< 0.0001
NIHSS above 8, baseline	−9.740	5.204	−0.143	−20.06 to 0.58	−1.872	0.064
mRS 3–5, baseline	−16.01	2.783	−0.453	−21.53 to −10.496	−5.755	< 0.0001
MoCA above 26, baseline	2.820	1.846	0.110	−0.540 to 6.481	1.528	0.130
**Dependent variable: Mobility domain** (ANOVA p < 0.0001); Adjusted R^2^: 0.452						
	**Coefficient B**	**SE**	**Standardized coefficients β**	**95%CI for B**	**t-value**	**p-value**
Constant	101.4	1.91		97.63–105.2	53.0	< 0.0001
HADS-anxiety 90 days after IS	−0.168	0.289	−0.058	−0.741 to 0.405	−0.581	0.563
HADS-depression 90 days after IS	−1.307	0.287	−0.454	−1.876 to −0.738	−4.554	< 0.0001
NIHSS above 8, baseline	−10.44	5.378	−0.155	−21.11 to 0.219	−1.942	0.055
mRS 3–5, baseline	−11.42	2.876	−0.328	17.12 to −5.713	−3.970	< 0.0001
MoCA above 26, baseline	0.794	1.907	0.032	−2.988 to 4.577	0.416	0.678
**Dependent variable: Social participation** (ANOVA p < 0.0001); Adjusted R^2^: 0.422						
	**Coefficient B**	**SE**	**Standardized coefficients β**	**95%CI for B**	**t-value**	**p-value**
Constant	89.9	3.649		82.67–97.14	24.6	< 0.0001
HADS-anxiety 90 days after IS	−0.082	0.551	−0.015	−1.175 to 1.011	−0.149	0.882
HADS-depression 90 days after IS	−2.250	0.547	−0.421	−3.334 to −1.165	−4.114	< 0.0001
NIHSS above 8, baseline	−13.53	10.25	−0.108	−33.86 to 6.794	−1.320	0.190
mRS 3–5 baseline	−19.87	5.481	−0.308	−30.74 to −9.002	−3.626	0.0004
MoCA above 26, baseline	7.985	3.635	0.171	0.766–15.194	2.196	0.030
**Dependent variable: Hand function** (ANOVA p < 0.0001); Adjusted R^2^: 0.489						
	**Coefficient B**	**SE**	**Standardized coefficients β**	**95%CI for B**	**t-value**	**p-value**
Constant	94.4	3.070		88.30–100.4	30.7	< 0.0001
HADS-depression 90 days after IS	−0.834	0.355	−0.164	−1.539 to −0.130	−2.347	0.021
NIHSS above 8, baseline	−23.95	9.164	−0.201	−42.12 to −5.783	−2.614	0.010
mRS 3–5, baseline	−29.39	4.910	−0.477	−39.13 to 19.66	−5.986	< 0.0001
MoCA above 26, baseline	7.948	3.261	0.178	1.482–14.414	2.437	0.016

Multiple regression analysis showed an association between living arrangements and the perception of some domains of HRQoL (Table 5). The mRS baseline was a significant negative predictor for 'Overall recovery,' 'Strength,' 'Mobility,' 'Hand function,' 'ADL/IADL,' and 'Social participation.' MoCA baseline was a significant negative predictor for 'Overall recovery,' 'Strength, ' 'Communication,' 'Social participation' and 'Hand function.' The HADS depression was a negative predictor of all domains of the SIS 3.0.

### Predictors of perceived social support

Multiple regression analysis identified only HADS depression after three months, and living arrangements as negative predictors for social support after IS (Table 6).

**Table 6:** Predictors of the MPSPSS Total score after three months. Results of multivariate linear regression analysis (model Enter)

**Dependent variable: MPSPSS Total scale** (ANOVA; p < 0.0001); Adjusted R^2^: 0.178						
	**Coefficient B**	**SE**	**Standardized coefficients β**	**95%CI for B**	**t-value**	**p-value**
Constant	6.514	0.162		6.192 to 6.836	40.1	< 0.0001
HADS-anxiety 90 days after IS	0.017	0.027	0.074	−0.036 to 0.070	0.634	0.528
HADS-depression 90 days after IS	−0.091	0.027	−0.397	−0.143 to −0.038	−3.404	0.001
Living arrangement (alone)	−0.366	0.172	−0.180	−0.708 to −0.025	−2.125	0.036
Age (under 50 years)	0.328	0.169	0.163	−0.006 to 0.662	1.943	0.054

## Discussion

This longitudinal observational cohort study explored the relationship between perceived adequacy of social support, functional outcomes, post-stroke symptoms, and HRQoL in young and working-aged adults three months after stroke. Furthermore, predictors of HRQoL in the initial recovery process in young stroke survivors were examined, including sociodemographic factors, post-stroke psychosocial symptoms, functional outcomes, and stroke-related variables. The perceived adequacy of social support as an independent variable was included.

On average, young stroke patients considered themselves to be well supported in the first months after IS. Perceived social support from family, friends, and significant others remained relatively stable over the first three post-stroke months. In contrast, perception of HRQoL, cognitive function, and functional outcomes increased significantly from baseline to three months.

Of concern, only a minority of the sample reported using and accessing of nursing or physiotherapy services after discharge from the hospital. Rehabilitation interventions are commonly provided within the first three months after stroke onset. However, findings from qualitative studies [29–31] showed that young stroke survivors perceived an absence of patient-oriented, age- and mild impairment-appropriate rehabilitation interventions supporting their transition from hospital or rehabilitation inpatient department to home within the first year after IS.

Participants’ perceived social support scores indicated they felt well supported by the three sources mentioned above (i.e., family, friends, and significant others). Furthermore, self-reported depression and anxiety symptomatology decreased over the FUP. A relatively stable level of perceived social support during the first months after stroke has been consistently found in observational studies [2, 21–24]. The present study did not consider the social network size, its elements, or the frequency of contacts. However, some studies have reported that despite a reduction in social networks after stroke, the perception of being supported remained unchanged [23].

Certain sociodemographic variables (living arrangements, age) were included as independent social support variables. Depression after three months and living arrangements (living alone) were negative predictors for perceived social support. To our knowledge, no previous literature has studied the perceived adequacy of social support as the dependent variable. Our study showed that social support did not significantly predict HRQoL domains. This finding contrasts previous reports [6, 13, 23]. In our study, most patients suffered from minor or mild IS, and most achieved excellent functional outcomes three months later (Table 1). Furthermore, patients in our study were substantially younger than those in previous studies [6, 23]. These facts may have contributed to our results. Research into the predictors of HRQoL has identified demographic factors, stroke-related factors, and social support as independent predictors [23]. However, many studies are limited by their cross-sectional design. Social support has mainly been investigated as an independent variable of HRQoL, associated with various other variables in multivariate analyses [6, 23]. Significant associations between social support and HRQoL have been reported in many studies [2, 16–18]. In addition, the relationship between specific dimensions (emotional support, informational support, tangible support, affectionate support, and social companionship) and sources of social support and HRQoL has been investigated. Associations between overall perceived social support and HRQoL were more significant than between specific dimensions or sources of social support and HRQoL [13]. Emotional support was a significant factor contributing to higher HRQoL using hierarchical multiple regression analysis in a previous study of the stroke population [41]. In a study focusing only on stroke survivors with chronic aphasia, social companionship and informational support were associated with better HRQL [2]. Moreover, combined sources of social support influenced post-stroke HRQoL more significantly than support from any single source [18].

The results of this study show a weak or moderate, albeit generally significant, correlation between functional social support and lower levels of depression and anxiety, stigma, emotional and poorly controlled behaviors, improved ability to participate in social activities, and greater satisfaction with social roles/activities over the first three months after IS. These findings are consistent with recently published meta-analyses and empirical studies indicating an inverse independent relationship between social support and post-stroke depression [42, 43]. Social support may reduce the negative impact of stigma and point to the degree to which stroke survivors feel supported, encouraged, and understood by society [44]. Significant associations between stroke-related stigma and social support have been confirmed in previous research [44, 45]. Higher levels of stigma were influenced by higher levels of depression, lower social support, lower functional ability, and stroke recurrence [44]. The present study did not find significant associations between perceived social support and post-stroke pain severity and interference. Predictors of HRQoL three months post-stroke were functional outcomes baseline and level of depression at three months. Furthermore, domain-specific predictors were also identified, such as living arrangement on emotions and memory, or HADS anxiety on emotions.

Several studies have noted the mediating effect of several variables on this relationship. Perceived social support for stroke survivors improved HRQoL by inducing positive rehabilitation motivation [43]. The potential mediating roles of social support and hope on HRQoL were explored in a longitudinal study using a three-wave structural equation model [16]. Hope was predictive in mediating the effects of functional impairment and social support on emotional distress and HRQoL [16].

Perceived adequacy of social support was not found to predict HRQoL at any time in our study. Inconsistent findings on the relationship between social support and HRQoL have been reported previously [13, 23], especially in young and working-aged adults [6], with only one study reporting a positive association [6]. However, we did not find any relevant associations. This might be because most of our patients had excellent functional outcomes after IS. Furthermore, patients in our study were substantially younger than those in previous studies. To our knowledge, no previous study has examined only working-age patients. We also used different study designs (longitudinal study), conceptualizations, and assessments of social support and HRQoL compared to previous studies. These findings raise several questions or suggestions for future research. Further research should focus on the relationship between perceived social support and HRQoL and involving patients with more severe strokes, residual neurological deficit and poorer functional outcomes.

## Study limitations

Our study has several limitations. Firstly, the relatively small sample size and single-center study design may limit the extrapolation of our results to the general stroke population. Secondly, the single-center study design cannot reflect the variability and availability of health care services in different regions and countries. Thirdly, most of our patients achieved excellent three-month functional outcomes; thus, the need for and impact of social support after IS may have been less relevant than expected.

## Conclusion

Living arrangements and post-stroke depression were found to be the predictors of social support after IS. Patients felt well supported by family, friends, and other significant familiars. The overall perceived support was not the predictor for any HRQoL domains. Functional outcomes and cognitive functions baseline and level of depression at three months were predictors of some domains of HRQoL. Focusing on the managing emotional problems and supporting functional outcomes may be modifiable factors representing targets for strategies to improve the HRQoL. Further research focusing on the relationship between perceived social support and HRQoL and involving patients with more severe strokes and poorer functional outcomes may help to understand better the role of social support in stroke recovery in working-aged patients.

## Data Availability

The datasets supporting the conclusions of this article are available from the corresponding author upon reasonable request. Data are not publicly available due to ethical reasons. Further inquiries can be directed to the corresponding author.
